# Short time-series microarray analysis: Methods and challenges

**DOI:** 10.1186/1752-0509-2-58

**Published:** 2008-07-07

**Authors:** Xuewei Wang, Ming Wu, Zheng Li, Christina Chan

**Affiliations:** 1 Department of Chemical Engineering and Material Science, Michigan State University, East Lansing, MI 48824, USA; 2 Department of Computer Science and Engineering, Michigan State University, East Lansing, MI 48824, USA; 3 Department of Biochemistry and Molecular Biology, Michigan State University, East Lansing, MI 48824, USA; 4 Biomedical Engineering Department, Boston University, Boston, 02215, USA

## Abstract

The detection and analysis of steady-state gene expression has become routine. Time-series microarrays are of growing interest to systems biologists for deciphering the dynamic nature and complex regulation of biosystems. Most temporal microarray data only contain a limited number of time points, giving rise to short-time-series data, which imposes challenges for traditional methods of extracting meaningful information. To obtain useful information from the wealth of short-time series data requires addressing the problems that arise due to limited sampling. Current efforts have shown promise in improving the analysis of short time-series microarray data, although challenges remain. This commentary addresses recent advances in methods for short-time series analysis including simplification-based approaches and the integration of multi-source information. Nevertheless, further studies and development of computational methods are needed to provide practical solutions to fully exploit the potential of this data.

## Background

Microarray technology has enabled the interrogation of gene expression data in a global and parallel fashion, and has become the most popular platform in the era of systems biology [1]. A majority of the microarray analysis thus far has focused on elucidating disease mechanisms [2]. More recently, with the rapid growth in research and development of biofuels [3], a new challenge of manipulating plant cell-wall biosynthesis has led to further applications of microarrays [3]. The detection and analysis of steady-state mRNA expression have become routine [4-7], with applications in many areas of biology (i.e., plants, yeast, insects, and mammals). Increasing efforts are focused on deciphering the multidimensional *dynamic *behaviours of complex biological systems, including complex regulation schemes, such as the crosstalk between multiple pathways [3,8,9], and interactions among more than 1000 genes in plant cell wall biogenesis, developmental biology, and human diseases [10-14]. Thus, time-series microarray data, and its analysis, are of growing interest to several research communities [15].

Time-series microarrays capture multiple expression profiles at discrete time points (i.e., minutes, hours, or days) of a continuous cellular process. These data can characterize the complex dynamics and regulation in the form of differential gene-expressions as a function of time. Numerous time-series microarray experiments have been performed to study such biological processes as the biological rhythms or circadian clock of *Arabidopsis*, flowering time, abiotic stress, disease progression, and drug responses [2,16-20]. Many of the methods of analyzing time-series data originated from various disciplines, such as signal processing, dynamic system theory, machine learning and information theory, and have been applied to detect differentially expressed genes, identify expression patterns, and construct gene networks [15,21-23], nevertheless challenges remain.

A significant challenge in dealing with time-series data comes from the limited sampling or number of time points taken, giving rise to short time-series data. In the growing pool of temporal microarray datasets, a typical time-series record has fewer than ten time-points [24]. The most common type of temporal data available is short time-series data, which arises from the difficulty in obtaining samples for many time points, often times due to the high costs of the arrays or limited biological samples, especially in animal or clinical studies [25,26]. "Short" time-series could signify the time-scale or the number of discrete time-points. Typically, it refers to the latter, which more appropriately should be *sparse *time-series data.

Limited sampling accentuates the difficulties associated with static or standard time-series analyses. First, the problems arising due to high dimensionality accompanied by a small sample size, such as matrix singularity and model over-fitting [27], in analyzing static or long time-series microarray data, become more pronounced in the case of short time-series data. Second, the unavoidable noise has more influence on the analysis of short time-series than on long time-series data, enhancing the difficulty in distinguishing real from random patterns and increasing the potential of misleading analyses [28].

**Figure 1:**
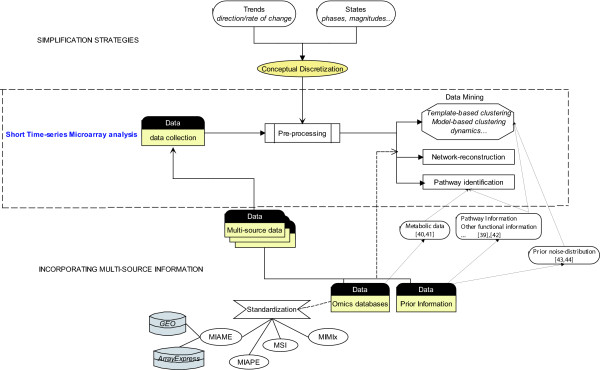
**The general process of *time-series expression analysis *starts with *data collection *from microarray experiments.** The data then undergoes *pre-processin*g procedures, such as normalization and quality evaluation. Next *data mining *techniques are used to *discover patterns or characteristics*, *identify related pathways *or *reconstruct systems network *for biological processes from short-time series data. To address the limited sampling in short-time series data, two strategies are introduced in the general process of microarray analysis. *Simplification strategies *reduce the data to discrete representations based on *trends *or *states *with respect to time to achieve more interpretable and biologically meaningful clusters. Such *conceptual discretization *is part of the pre-processing step, prior to data mining. *Incorporating multi-source information *takes a different strategy. In this strategy *multi-source data*, including various *omics databases *and *prior biological information*, are collected and integrated to obtain a comprehensive dataset and enhance the information content. To minimize the heterogeneity of *omics data *from different experiments, *standardization *can and have been imposed on *omics databases*. Current standards for high-through-put database include *MIAME, MIAPE, MSI, MIMIx*. MIAME has been implemented with *GEO *and *ArrayExpress *microarray databases. The integration of various *omics databases *or *prior biological information *can enhance the effectiveness and efficiency of mining and interpretation of short-time series data to achieve biological discoveries. For example, multi-source *prior biological information*, i.e., *prior noise-distribution *has been proposed to enhance the performance of the data mining and network inference [43,44]. In addition, *pathway and functional knowledge *and *metabolic data *from different databases have also enhanced the clustering results and pathway identification [39-42]. These studies are discussed and referenced in the text.

Improving short time-series analysis requires addressing the problems that arise due to limited sampling. Recent efforts by investigators to overcome the difficulties associated with limited sampling include decreasing the complexity of continuous time-series data based on simplification strategies [29,30] or enriching the information content of the data by incorporating multi-source information [31,32], see Figure 1 for a summary of possible options.

## Simplification strategies

Simplification strategies reduce time-series data from continuous to discrete representations prior to analysis. These strategies usually transform the raw temporal profiles into a set of symbols [29,30,33] or nominal values [31,34] that are used to categorize qualitatively the gene expression data into different states or trends, that is, in terms of phases (early or late), magnitudes (high or low), or directions (up- or down-regulation). Based on this concept, a "quantization" method introduced by Di Camillo et al[35], whereby the expression of a gene at a particular time-point is quantized (discretized) into three patterns of "states", representing under-expressed, not differentially expressed or over-expressed with respect to a baseline pre-defined by a hypothetical distribution. After such discretization, the Dynamic Bayesian Network algorithm performed better in terms of precision and recall in reconstructing the regulatory network from synthetic expression data generated from differential equations based on a series of defined rules of regulation. Similarly, Kim [33] developed a difference-based clustering algorithm (DIB-C) in which the profile of short time-series data was discretized to symbolic patterns, but according to the differences between adjacent time-points. These patterns or "trend" simplified the profile of a gene from numerical values to direction of change, that is, "*I *(Increase), *D *(Decrease) or *N *(No change)", and rate of change, that is, "*V *(conVex), *A *(concAve) or *N *(No change)". Inevitably information is lost through this simplification. Even so, such conceptual discretization helped achieve more interpretable and biologically meaningful clusters [33].

Simplifications methods have a side benefit in reducing the noise in the original data to some degree when decreasing the dimension of the time-series data, thus making the subsequent analysis more robust to noise. This was demonstrated by Sacchi et al. [30] with their adaptation of the Temporal Abstractions (TA)-clustering method from the field of artificial intelligence to gene expression analysis. Here, the temporal expression profiles were described in terms of trends of "Increasing", "Decreasing", or "Steady". A reduced rate of misclassification in computational experiments was observed for simulated data using TA-clustering with pre-defined patterns and noise than with the clustering approach without such simplification strategies, particularly when the noise level was high [30].

A key challenge with simplification strategies is how to pre-define these *a priori *representative temporal trends or patterns of gene expression in the discretization step. Defining these patterns have largely depended on the expertise of the researchers, for example, Gerber et al defined six temporal expressions trends in terms of phase (early, middle and late) and direction (increase and decrease) [31], similarly, Wu et al. proposed 27 possible temporal patterns to group gene expression data for CD8 T cell differentiation [34]. However, this may introduce bias in the patterns that are pre-defined and, in turn, the analysis and results obtained. Data-driven approaches could extract potentially novel gene expression patterns in an objective and reasonably unbiased fashion [36]. Thus, developing methods to automatically define temporal trends could alleviate this limitation or bias. Ernst et al. proposed a procedure to generate potential trends which describe the directions and magnitudes of the expression changes with respect to time [24,28]. Attempts at automatic abstraction of temporal features have met with some success in providing easily interpretable clusters, examples include the temporal abstraction-based method that defines trends (i.e., *Increasing, Decreasing and Steady*) over subintervals [30], and the difference-based method that uses the first and second order differences in expression values to detect the direction and rate of change of the temporal expression [33]. Although simplification strategies make the raw expression profiles coarse-grained, which could somewhat ameliorate the noise in the data, inevitably the simplification leads to loss of information, which may exacerbate the situation of limited sampling. In particular, some important patterns may be lost when the raw expression profiles are oversimplified, for example, simplifications that consider only monotonously expressing genes [31] may not capture some of the complex temporal patterns, such as oscillatory gene expression profiles [37].

## Incorporating multi-source information

Incorporating multi-source information, including prior knowledge (i.e., pathway information) [38,39], multi-scale or different levels of information [40-42], or additional time-series datasets from other sources [31,32], is another approach to address the limited sampling and to improve the computational analysis and interpretation of short time-series microarray data.

Different types of prior knowledge have been used to improve the computational analysis of short time-series data. They include applying a prior noise distribution to the expression data [43]. For example, by incorporating a prior noise-distribution to improve the parameter estimation in the commonly used CAGED model (Cluster Analysis of Gene Expression Dynamic), Wang et al. achieved more functional and meaningful clusters, as validated by Gene Ontology [43]. This approach was advanced further by Wang et al. [44] to a stochastic dynamic model where the gene expression profile is modelled with the addition of noisy "measurements". The authors try to explicitly separate the real pattern of expression from the Gaussian noise imposed onto the expression data. Based on such a model, they applied Expectation Maximization (EM) algorithm to estimate both the parameters for the noise model and the actual values of the expression levels, and efficiently reconstructed the gene regulatory network. Thus defining a prior noise-distribution in analyzing time series microarrays is both biologically relevant and computationally efficacious especially when the time series is too short to satisfy the requirements of traditional multivariate methods for parameter estimation [44].

In addition, pre-defined gene sets involving specific pathways or functional categories have focused on pattern changes of sets of genes rather than individual genes and helped to enhance our understanding of cellular processes [38,39]. Similarly, incorporating multi-level biological information, such as metabolic data or prior knowledge about the genes and pathways, has improved interpretation of the data. For example, metabolic data [40,41] and pathway information [40,42] have been integrated with short time-series gene expression data to identify liver toxicity pathways in HepG2 cells. Likewise, protein-DNA interaction data and promoter motif information have been integrated with short time series data to reconstruct the dynamic gene regulatory network of *Saccharomyces cerevisiae *response to stress [45], and to identify targets of known transcription factors in cold acclimation of *Arabidopsis thaliana *[46], respectively. Furthermore, metabolic profiles have been integrated with short time-series gene expression data to characterize the dynamics of metabolic changes during oxidative stress [47], the effect of elevated CO_2 _on the physiology of *A. thaliana *[48], and to reconstruct the temporal sequence of events during bud development [49]. Similarly, integrating multiple time-series datasets has become increasingly popular with the growing pool of publicly available datasets [50]. Combining multiple time-series datasets has been shown to improve the confidence of the gene regulatory relationships that are inferred [51], as well as identify regulatory relationships [32] and functional gene clusters [31] under different treatment conditions.

A key challenge with integrating different datasets is the heterogeneity of the data, that is, each set may have a unique set of sampling rates, time-scales, cell types, and sample populations, as well as varying measurement noise levels, etc. The heterogeneity across the datasets increases the difficulty in extracting meaningful results. To maximize the usefulness and minimize the heterogeneity of the publicly available data, stricter standardization methods should be defined and imposed on procedures such as data collection and pre-processing. Indeed, standards such as MIAME (Minimum information about a microarray experiment), MIAPE (Minimum information about a preoteomics experiment), MSI (Metabolomics standards initiative), MIMIx (Minimum information required for reporting a molecular interaction experiment) have been proposed and implemented for presenting and exchanging gene expression [52], proteomics [53], metabolomics [54] and interaction data [55], respectively. Thus far, standardizing gene expression data is the most mature and hence, most successful compared to the standardization of the other data types. Therefore, integrating gene expression data from various sources is now readily achievable with public databases, such as GEO [56] and ArrayExpress [57], where the quality of the data is controlled with the MIAME score.

## Conclusion

In summary, analysis of short time-series microarrays is still at an early stage. Most studies using short time-series data have applied methods that had been developed for static or long time-series microarray data, and which tend to perform poorly with limited temporal sampling. Current efforts, including simplification approaches and the integration of multi-source information, have shed promising light on improving the analysis of short time-series microarray data.

Future studies could combine both of these strategies to simultaneously decrease the complexity of continuous time-series representations, yet minimize the information loss with the simplification-based approaches by increasing the information content of the data. Gene-module-level analysis could be a potential solution, in which the concept of modularity not only plays a central role in incorporating multi-source biological information, but also reflect a simplification strategy focusing on groups of genes rather than individual ones. Gene-module-level analysis could efficiently combine both strategies.

A recent study by Hirose et al [58] used a statistical inference method to reconstruct a module-level gene network based on time-series data, rather than networks of individual genes. They concentrated on groups of genes and the correlations between them, thus the transcription modules extracted could be building blocks of the regulatory networks. Such module-based network construction overcomes, in part, the problem of limited sampling. The modules in the study are calculated by a vector regressive approach based on the state space model, which essentially simplifies the data by including only the significant temporal relationships between the modules. Unfortunately, their modules are defined based on statistical criteria and thus are limited in their biological significance. The integration of multi-source biological information to identify modules from short-time series microarray data should enhance understanding and interpretation of biological systems and disease processes.

Thus far, the predominant focus has still been on lower levels of analyses, such as detecting differently expressed genes or clustering genes with similar temporal profiles, whereas few higher levels of analysis, i.e. network construction, have been reported. With the rapid growth in availability of short time-series data, more theoretical and technical studies are urgently needed to provide practical solutions to exploit fully the potential of this wealth of data.
